# Scoring System Assessment of Cephalic Vein Access for Device Implantation

**DOI:** 10.19102/icrm.2018.090802

**Published:** 2018-08-15

**Authors:** Jane Taleski, Lidija Poposka, Filip Janusevski, Bekim Pocesta, Vladimir Boskov, Noel G. Boyle

**Affiliations:** ^1^Department of Electrostimulation and Electrophysiology, University Clinic of Cardiology, Skopje, R. Macedonia; ^2^University of California Los Angeles Cardiac Arrhythmia Center, Los Angeles, CA, USA

**Keywords:** Axillary vein, cephalic vein, device implantation, score assessment, subclavian vein

## Abstract

The purpose of this study was to explore the usability of the cephalic vein (CV) for cardiac implantable electronic device (CIED) lead access by applying a scoring system to assess the venous anatomy. This prospective, single-center study included 100 consecutive patients who underwent CIED implantation within a period of one year. Contrast-enhanced venography images were obtained for every patient, focused on the CV, “T-junction,” and the subclavian/axillary veins (SV/AVs). Though careful examination of the images, an angle, valves, diameter, noncollateral (AVDnC) score was constructed and used to aid in choosing a CV or SV/AV access approach; in all cases, however, the preferred approach was CV independent of the AVDnC score result obtained. Upon use of the scoring system, the majority of patients (54%) had type A score result (≥ 3), indicating a favorable anatomy for CV access. In 48 of these patients, the CV was used for the implantation of at least one lead. The remaining 46 (46%) patients had type B score result (≤ 2). In 41 patients from this group, SV/AV access was used for lead implantation and, in five patients, CV access was used. The number of leads introduced through the CV was associated with larger score and the operator’s experience. In conclusion, in more than 50% of patients, at least one lead could be introduced through the CV. The scoring system used herein can simplify the choice between CV and SV/AV access and could eventually increase the efficiency and safety of the procedure, especially when less experienced implanters are involved.

## Introduction

The number of procedures for cardiac implantable electronic device (CIED) placement continues to increase.^1^ Many reports have examined variations of CIED implantation techniques.^2–4^ Of course, no account can totally cover all of the implant techniques and variations used by different operators; as with any practical skill, written descriptions cannot replace the practical experience of a skilled implanter.^2^

A CIED implantation technique can be divided into several stages. The first stage begins by attaining central venous access for lead insertion. The most commonly used techniques are the cephalic vein (CV) “cut-down” method and direct needle puncture of the subclavian vein (SV) or axillary vein (AV) (ie, the Seldinger technique).^3,5^ The CV is located in the deltopectoral grove, penetrating the clavipectoral fascia to join the axillary vein medial to the pectoralis minor muscle and then together continuing as the subclavian vein.^2^ Variations and anomalies of the CV may prolong the procedure time and result in unsuccessful cephalic access in up to 40% of patients, especially for less experienced operators. Improvements to this technique have been proposed, such as using a hydrophilic guidewire in difficult cases, which helped to decrease failure rates to 10% to 20%.^6,7^ The SV/AV approach can be used as a first or alternative choice, especially because of a more rapid learning curve. Less experienced implanters are usually more confident with SV puncture, particularly if an extrathoracic approach is used.^8^ In both techniques, it is highly recommended to have fluoroscopic guidance, especially for axillary and intrathoracic SV puncture.^4^ These approaches can result in complications including pneumothorax, hemopneumothorax, inadvertent subclavian artery puncture, brachial nerve plexus injury, thoracic duct injury, and mechanical damage to the lead(s).^9^

In this study, a scoring system for CV assessment was developed and evaluated, based on a venogram performed before each access attempt.

## Methods

This single-center, prospective study included 100 consecutive patients referred for CIED implantation between March 2015 and March 2016. This study was approved by the hospital’s ethics committee. The first choice for central venous access for every patient included was the CV cut-down technique, regardless of the angle, valves, diameter, noncollateral (AVDnC) scoring result (ie, A or B), before attempting SV/AV access. This technique was explored by extrathoracic fluoroscopy-guided SV puncture or, alternatively, by contrast venography-guided AV puncture. With respect to devices implanted, the study included mostly single/dual-chamber permanent pacemakers (PPMs) along with several dual-chamber implantable cardioverter defibrillators (ICDs) and one cardiac resynchronization therapy pacemaker (CRT-P) device. Procedures were performed via both left- and right-sided approaches under local anesthesia. Antibiotic prophylaxis was used routinely. All procedures were performed by experienced operators (with a record of more than 100 CIED implants per year and more than five years of experience with CIED implantation). The center complication rate was 1.5% per year.^10,11^

During the first stage of every procedure, before local anesthesia, 10 mL of undiluted contrast was injected (Ultravist^®^ 370; Bayer AG, Leverkusen, Germany) through the left/right antecubital vein. In posteroanterior projection, contrast-enhanced venographic images were made of the CV in the deltopectoral triangle (ie, between the site of lead insertion and the T-junction with the AV). We focused our attention on the CV, its entrance in the AV, the T-junction, and the SV position and anatomy. By postprocedural morphometric analysis of the CV venogram, AVDnC score was calculated and used to classify the venous anatomy as type A (score ≥ 3) or type B (score ≤ 2). The cutoff number was carefully made by combining the most frequent/favorable scoring variables for lead introduction. This scoring system was then used to grade the ease of using the CV versus the SV/AV access technique; however, our priority approach was CV, irrespective of the score.

**Figure 1:**
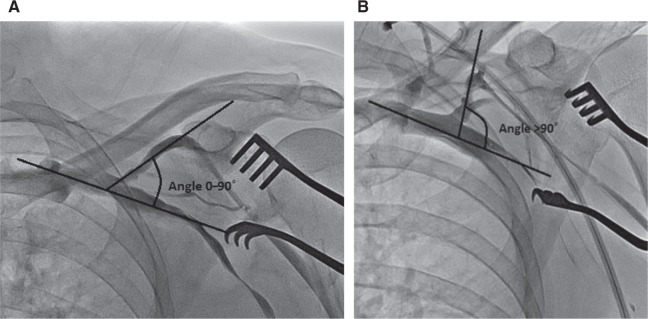
Angle measurement for the cephalic/AV junction. **A:** Angle measurement of 0 degrees to 90 degrees (“A” from the AVDnC score). **B:** Angle measurement of > 90 degrees (“A” from the AVDnC score).

The AVDnC score was constructed after careful examination of all 100 patient venography images. All four variables were connected with the most often-seen morphometric varieties of the CV region during this study **(Table 1)**. The first variable of the score (angle) refers to the angle of the T-junction between the CV and the AV. We gave 2 points for an angle of between 0 degrees and 90 degrees pointing medially **(Figure 1A)**, and 0 points for all other angle presentations in this study **(Figure 1B)**.

The second variable (valves) refers to vessel valves; it assesses the presence or absence of the valves in the CV lumen, especially in the place where the CV joins the AV. One point was assigned for one or no valves present in the CV lumen or in the region of the T-junction, while 0 points were assigned to all other patients.

The third variable (diameter) refers to the diameter of the CV. One point was allocated for a CV diameter greater than or equal to one-third the diameter of the SV, and 0 points were allocated for other presentations.

**Figure 2:**
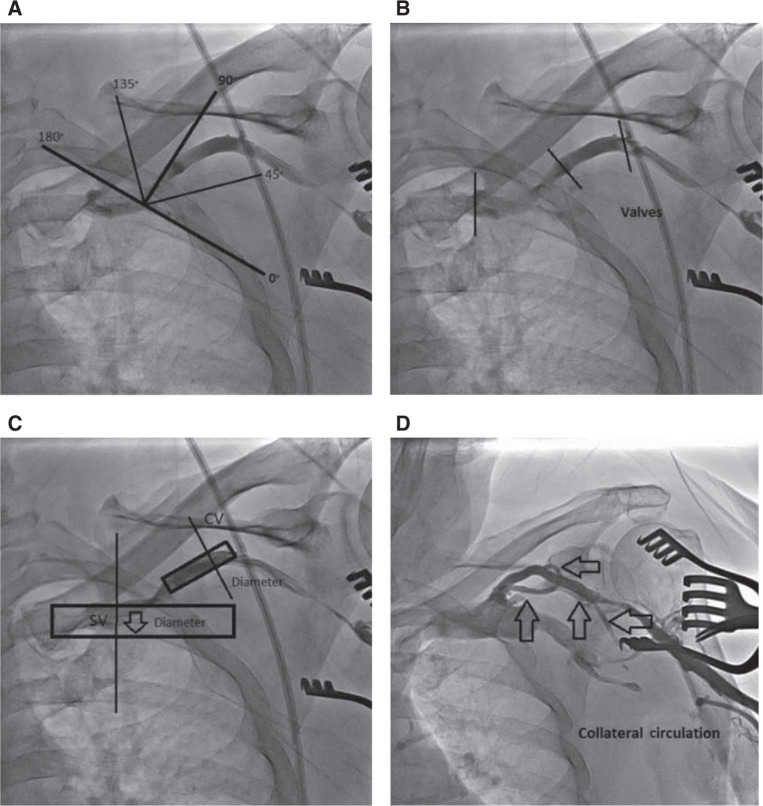
How the AVDnC scoring system works. **A:** Angle. **B:** Valves. **C:** Diameter. **D:** Noncollateral.

The fourth and last variable (noncollateral) refers to the absence of collateral venous circulation around the CV or the parallel additional vein that usually also joins with the AV. One point was designated for patients who had a single CV without any collateral circulation, and 0 points were designated for all others **(Figure 2)**.

**Figure 3:**
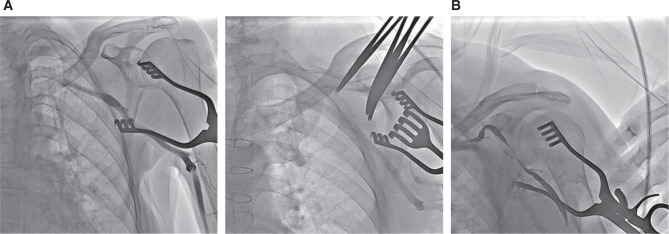
AVDnC scoring result Type B. **A:** Type B score of 0 points (no CV/low entrance). **B:** Type B score of 2 points.

The scoring system also included patients with a CV that was not visible when performing venography, most commonly due to an CV–AV confluence site that is decidedly lower than the expected region.^1^ This type of CV patient was given 0 points **(Figure 3A)**.

**Figure 4:**
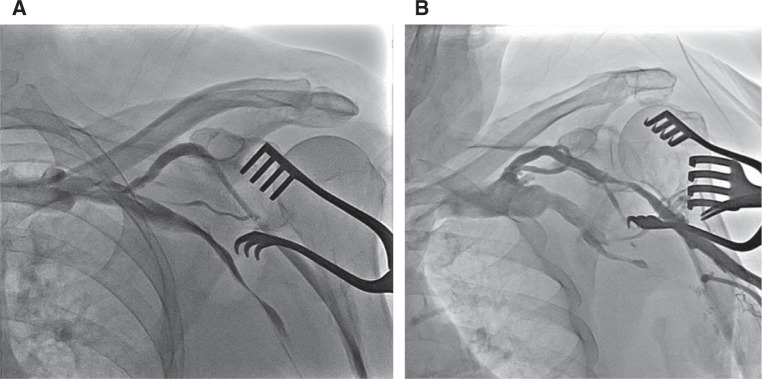
AVDnC scoring result type A. **A:** Type A score of 5 points. **B:** Type A score of 3 points.

After a thorough evaluation of these variables, patients were divided into two groups. Type A included patients who had a total score of three or more (≥ 3) **(Figures 4A and 4B)** and type B included those patients who had a score of less than three (≤ 2) **(Figures 3A and 3B)**. Type A patients were classified as good candidates for introducing one or two leads through the CV.

For both patient groups (those with type A and those with type B scores, respectively), we used CV access as the first choice and SV/AV as the second choice for venous access. At the end of every procedure, fluoroscopic images were stored of the deltopectoral groove showing the pacemaker generator and pacing leads.

We also measured the time of the first stage of every procedure, starting from the time of local anesthesia injection until the introduction of the leads, irrespective of type A or type B group or whether the CV or SV/AV was used as the final central venous entrance site.

### Statistical analysis

Categorical variables are expressed as numbers and percentages and continuous variables are expressed as the mean ± standard deviation or median (interquartile range). Statistical analysis was performed using the Statistical Package for the Social Sciences software version 17.0 for Windows (IBM Corp., Armonk, NY, USA).

## Results

During a period of one year, 100 venography-guided CIED implant procedures were evaluated. Sixty-seven (67%) patients were male and 33 (33%) were female. The mean age of the patients was 71 years ± nine years. Ninety-six (96%) CIEDs were implanted from the left side and four (4%) CIEDs were implanted from the right (deltopectoral) side.

During this period of time, 96 (96%) of the CIEDs implanted were PPMs. This group included 41 dual-chamber pacemakers (DDD), seven single-chamber with atrial sensing coil pacemakers (VDD), and 48 single-chamber pacemakers (VVI). The study also included three (3%) dual-chamber ICDs and one (1%) CRT-P device. The pacing/defibrillation leads were implanted using active fixation in 95 (95%) patients and using passive fixation in five (5%) patients.

**Figure 5:**
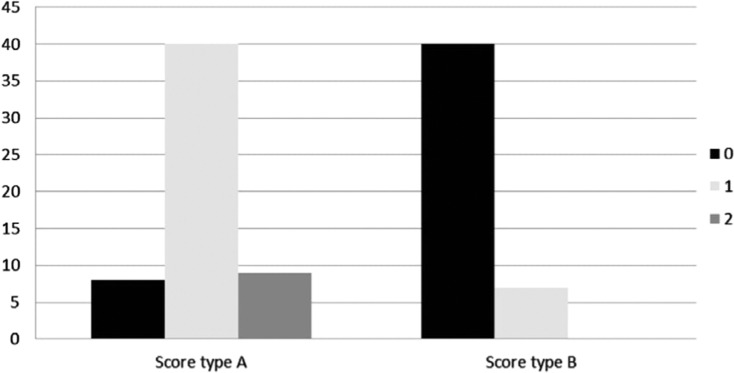
Number of leads placed through the CV (either zero, one, or two) versus CV type (A or B).

The majority of patients in this study (54%) had a score of ≥ 3 (type A). In 48 of these individuals, the CV was used for implantation of at least one lead. This group was further divided into two subgroups: a larger one that included 31 patients with a score of 4 or 5 (seven with two implanted leads through the CV) and a smaller one of 23 patients with a score equal to 3. In both subgroups, the implantation of one or two leads via the CV was achieved in all patients. The remaining 46 (46%) patients were type B with a score of ≤ 2. SV/AV access was used for lead implantation in 41 patients from this group, and CV access was used in five patients **(Figure 5)**. This group included the 25 patients with no visible CV identified while performing venography. In six of these patients, the CV–AV confluence site was below the expected location.

The scores of the individual AVDnC variables were also evaluated. With respect to the first variable (angle), 56 of 100 patients garnered 2 points and, in 49 of these individuals, at least one lead was introduced through the CV. Suitable (one-third of the SV) vessel diameter was present in 59 patients. Forty-five patients had more than one valve and the CV was successfully used in 18 of them. Regarding the last variable, 23 of 100 patients presented with collateral circulation. These results showed the importance of each variable—especially the angle and diameter variables—in patients in whom the CV approach was used.

**Figure 6:**
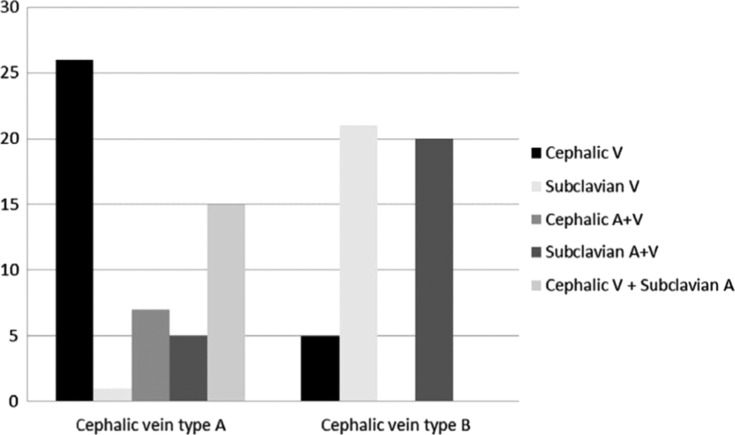
Lead access versus CV type (A or B).

In summary, we were able to easily introduce at least one lead through the CV in more than 50% of all 100 patients; however, the number of introduced leads through the CV is not only related to a larger score but may also depend on the operator’s experience **(Figure 6)**.^10,11^ We were not able to use the CV for access in six patients with type A score; instead, the SV/AV was used for lead insertion. CV access was easily employed in five patients with a type B score **(Table 2)**. Procedure time (the time spent to gain central venous access and introduce one lead) was measured in all cases in both groups from the first stage of the procedure (the injection of contrast) until the first lead was introduced into the access vein. Pocket formation time was not included. In patients with type A in whom the CV was used, the time frame was from eight minutes to 10 minutes; this was similar to the time period in type B patients in whom SV/AV access was used, which was six minutes to nine minutes. However, in some patients with type B score in whom we tried to use the CV, the access time ranged from 20 minutes to 25 minutes and, in some patients, we were obligated to switch to SV/AV access.

## Discussion

Effective introduction of CIED therapy requires successful central venous lead access.^12^ The CV cut-down technique is usually the primary choice of many operators for introducing leads into the central venous system and has advantages over SV/AV puncture techniques.^3,5^ However, successful lead passage through the CV depends on the morpho-anatomical parameters of the vessel and other factors such as the size and structure of the leads introduced via the CV, vessel wall elasticity, and operator experience.^1,9,13^ In this study, CV access was achieved in 48 of 54 (88.9%) of those individuals with a favorable score (type A) but only in five of 46 (10.9%) of those with an unfavorable score (type B). The morpho-anatomical characteristics of the CV were analyzed and related with successful lead passage in one prior study.^1^ In that study, contrast venography was performed and the results were obtained during first-time lead placement only in the cases of problematic lead introduction (15%) with either the CV cut-down or SV/AV puncture techniques analyzed. Unfavorable morpho-anatomical parameters were CV diameter ≤ 1 mm (18%) and the presence of sharp curvature of the terminal CV segment as it joined the axillary vein (14%).^1^

Another prospective study showed a 17% failure rate in lead placement through the CV approach was connected with small vessel size.^13^ In addition to the previously mentioned CV morpho-anatomical parameters, one study described a technique where ultrasonography was used for the evaluation of the vessel prior to the procedure to minimize the failure rate.^14^ Different studies have described variations to the classical CV cut-down technique, including the use of a hydrophilic guidewire, cannulation of retropectoral veins, the simultaneous use of two guidewires, and the use of stiff angiography guidewires. These “no puncture” approach techniques proved successful in more than 95% of implantations and with an absence of major complications.^6,7,15^ Because of the rapid learning curve, the SV/AV puncture technique is widely used and less experienced implanters are usually more confident employing this approach. However, this technique can lead to serious complications, including pneumothorax, hemopneumothorax, inadvertent subclavian artery puncture, brachial nerve plexus injury, thoracic duct injury, and mechanical damage of the leads.^9^

One prospective randomized study compared the safety and effectiveness of the placement of endocardial pacemaker and defibrillator leads using the extrathoracic SV guided by contrast venography versus the cephalic approach and found that this approach was safe and efficient. It was also associated with no increased risk of complications as compared with the CV approach and can be used as an alternative to such.^8^

### Limitations of the study

This is a single-center study involving a limited number of operators and patients. Larger numbers of patients and operators are needed to further validate the scoring system described in this study.

## Conclusion

The scoring system we have introduced can simplify the selection of appropriate patients to undergo the CV approach, possibly facilitating the performance of this technique especially for less experienced implanters and eventually increasing the efficiency and safety of the procedure.

## Figures and Tables

**Table 1:** AVDnC Scoring System

**A (angle)**	
0–90 degrees	2 points
> 90 degrees	0 points
**V (valves)**	
No or one valves	1 point
More than one valve	0 points
**D (diameter)**	
CV diameter greater than or equal to one-third that of the SV	1 point
All others	0 points
**nC (non-collateral)**	
Single CV without any collateral circulation	1 point
All others	0 points

AVDnC: angle, valves, diameter, non-collateral; SV: subclavian vein; CV: cephalic vein.

**Table 2:** Patient Characteristics

Patient Characteristic	Type A	Type B	p-value
Number, n	54	46	
Male gender, n (%)	36 (67%)	32 (69%)	0.463
Age	70.41 ± 9.39 years	74.56 ± 7.44 years	0.537
Lead access			
CV (ventricular lead position), n (%)	26 (48%)	5 (11%)	< 0.003
Subclavian vein (ventricular lead position), n (%)	1 (2%)	21 (46%)	
CV (ventricular lead and atrial lead positions), n (%)	7 (13%)	0	
Subclavian vein (ventricular lead and atrial lead positions), n (%)	5 (9%)	20 (43%)	
CV (ventricular lead position) and subclavian vein (atrial lead position), n (%)	15 (28%)	0	
Calculated score			
0 points	1 (2%)	24 (52%)	< 0.003
1 point	0	5 (11%)	
2 points	0	17 (37%)	
3 points	23 (43%)	0	
4 points	19 (35%)	0	
5 points	11 (20%)	0	
Number of leads introduced through the CV			
0 leads	6 (11%)	41 (89%)	< 0.003
1 lead	41 (76%)	5 (11%)	
2 leads	7 (13%)	0	

n: number; CV: cephalic vein; SV: subclavian vein.
